# Spatial properties of odd and even low order harmonics generated in gas

**DOI:** 10.1038/srep07786

**Published:** 2015-01-14

**Authors:** G. Lambert, A. Andreev, J. Gautier, L. Giannessi, V. Malka, A. Petralia, S. Sebban, S. Stremoukhov, F. Tissandier, B. Vodungbo, Ph. Zeitoun

**Affiliations:** 1 Laboratoire d'Optique Appliquée, UMR 7639, ENSTA-CNRS-École Polytechnique, Chemin de la Hunière, 91761 Palaiseau, France; 2 Unità Tecnica Sviluppo di Applicazioni della Radiazione — Modellistica Matematica, ENEA Centro Ricerche Frascati, Via Enrico Fermi 45, 00044 Frascati, Italy; 3 Faculty of Physics, Lomonosov Moscow State University, Leninskie Gory, 1, build.2, 119991, Moscow, Russia; 4 National Research Centre “Kurchatov Institute”, pl. Akademika Kurchatova, 1, Moscow, 123182 Russia

## Abstract

High harmonic generation in gases is developing rapidly as a soft X-ray femtosecond light-source for applications. This requires control over all the harmonics characteristics and in particular, spatial properties have to be kept very good. In previous literature, measurements have always included several harmonics contrary to applications, especially spectroscopic applications, which usually require a single harmonic. To fill this gap, we present here for the first time a detailed study of completely isolated harmonics. The contribution of the surrounding harmonics has been totally suppressed using interferential filtering which is available for low harmonic orders. In addition, this allows to clearly identify behaviors of standard odd orders from even orders obtained by frequency-mixing of a fundamental laser and of its second harmonic. Comparisons of the spatial intensity profiles, of the spatial coherence and of the wavefront aberration level of 5*ω* at 160 nm and 6*ω* at 135 nm have then been performed. We have established that the fundamental laser beam aberrations can cause the appearance of a non-homogenous donut-shape in the 6*ω* spatial intensity distribution. This undesirable effect can be easily controlled. We finally conclude that the spatial quality of an even harmonic can be as excellent as in standard generation.

High harmonic generation in gas results from the strong non-linear polarization induced on a gas medium by focusing of a linearly polarized laser field to an intensity of about 10^14^ W.cm^−2^. The mechanism, which gives birth to the harmonic emission, can be described in the three steps model approximation. Microscopically, every half optical cycle, electrons are snatched out of their parent atom, accelerated in the intense driving electric field, and then accelerated back to the atom when the electric field reverses. When recombining with the atom core the electrons liberate their accumulated kinetic energy as an attosecond burst of photons. This gives rise to the emission of a series of fully coherent harmonics, only odd orders, spanning a wide range of the electromagnetic spectrum from the near Ultra-Violet (UV) to the soft X-rays. Two major electrons trajectories, called the short and the long ones, contribute to the generation of these harmonics.

The harmonic generation methods have been intensively developed during the last 20 years with similar underlying motivations: higher energy per pulse^1^,^2^,^3^,^4^, shorter pulse duration^5^, higher repetition rate^6^ and shorter wavelength^7^,^8^,^9^. Actually, the continuous improvement of this source is turning it, year after year, into a competing candidate with conventional facilities delivering short wavelength radiations to users, like synchrotrons and Free-Electron Lasers (FEL), especially in specific applications not particularly demanding in terms of photon flux. The high repetition rate (up to tens of MHz), associated to the femtosecond (fs) pulse duration, and the fact that no jitter occurs between the fundamental laser and the harmonics is a unique combined feature. This allows to distinguish these harmonics with respect to the other available sources in a similar spectral range. Recently, intense harmonics with quasi-circular polarization have even been produced efficiently^10^,^11^,^12^.

Several techniques have been elaborated to increase the generation efficiency, and to further extend the spectral range to shorter wavelengths, but although important progresses have been made, these two aspects still engender a limitation for quite a few applications. One of these techniques consists in the generation of harmonics with mixing of two frequencies: the fundamental laser (*ω*) and its second harmonic (2*ω*)^13^,^14^,^15^. In this case the increase of generation efficiency, especially at short wavelengths, is accompanied by the simultaneous generation of even order harmonics, further extending the spectral range of emission. Yet, as far as we know, a comprehensive study of the properties of isolated harmonics -and especially of the discrimination between odd and even orders- has never been conducted. In this paper, we report for the first time a detailed study of one of these even harmonics, 6*ω* at 135 nm (9 eV), spectrally isolated from contamination of surrounding odd harmonics, using interferential filters. Indeed, at low orders, the natural wavelength separation from the contiguous odd harmonics simplifies the diagnostics of the emitted light and helps to the characterization of the process of even harmonics generation.

It is commonly admitted that mechanisms of high and low order harmonic generation are different, and especially the three steps model is typically considered to be valid only for the description of high harmonics. Different methods were used to explain the generation of the harmonics at low orders, the so-called below (and near) threshold harmonics, which photon energies are less than (comparable to) the atom ionization potential^16^,^17^,^18^,^19^,^20^,^21^. In most cases, the short trajectory dominates the output because its phase is less sensitive to the driving laser intensity. However, it is still not clear whether the short trajectory exists in below threshold harmonic generation. In addition, excited state resonances may play a major role in below threshold harmonic generation. Therefore, there are major differences between below and above harmonic generations. Yet, their origins can be unified. First, while the three steps model used for high orders is not directly applicable to this spectral region, it has been generalized to qualitatively understand the process^18^,^19^. The second way relies on the unified non-perturbative full quantum-mechanical theory of harmonic generation^21^. The theory describing both high order and below threshold harmonic generation was developed in ref. 21, 22. As a consequence, part of the conclusions that are drawn from this study on low order harmonics^23^,^24^,^25^,^26^,^27^ are then potentially valid for the generation of higher orders.

Moreover, low order harmonics are almost unexplored in term of applications^28^,^29^ while numerous of them can already be performed taking advantage of their high spatial and temporal coherence properties. Seeding a FEL is one of the most promising applications. The intrinsic shot-noise in the electron beam, which is the driving source of synchrotron light emission, scales with the emission energy. In UV region, the available flux from harmonics in gas is then largely sufficient to transfer its coherence properties to the beam. Developing and studying harmonics of Ti:Sa lasers (800 nm) in the spectral range between typically 2*ω* (400 nm) and 7*ω* (114 nm) is consequently essential for the continuous progress on seeded FEL. Experiments, in which 5*ω* generated from gas target is amplified directly by the FEL medium (SCSS^30^ and SPARC^31^), have already been performed or are planned to be done (MAX-IV^32^). Amplification in cascade of wavelengths (SPARC^33^ and FERMI^34^) to get emission at short wavelengths have also been carried out more lately but are limited to the seeding of nonlinear harmonics from crystals, as intense injection signal is requested. Also, it is likely that plasma-based FELs^35^ would be demonstrated in seeding configuration at low orders for which the FEL gain is sufficiently strong. Photoemission spectroscopy^36^,^37^,^38^, a technique for which nonlinear-crystal based sources are requested so far, could also benefit from this low order harmonic source from gas which is now competitive and in particular at higher frequencies than 5*ω*, where the generated radiation is absorbed by the crystal itself. More generally, most of the applications of harmonics such as imaging experiments of ultrafast phenomena in matter at nanometer-scale require intense and diffraction-limited soft X-ray beams^39^, and therefore spatial properties of harmonics are of paramount importance.

We present here the measurements of the harmonic generation efficiency, together with the spatial intensity profiles, the spatial coherence, and the spatial aberrations of low order harmonics. Precisely, we compare for each measurement the 6*ω* radiation at 135 nm obtained by frequency mixing, the two-color configuration (*ω* + 2*ω*) case, and the 5*ω* radiation at 160 nm obtained in the classical way, the single-color configuration (*ω*) case. These information will contribute to characterize the process of even harmonic generation and appreciate its potential for applications. Finally, we also establish that fundamental laser aberrations (here essentially astigmatism) can cause the intensity distribution of the second harmonic beam to be non-Gaussian at the focal spot inside the gas target, creating in our case a non-homogeneous donut shape intensity distribution with two main lobes in the far-field of the 6*ω* beam.

## Results

### Harmonic generation efficiency

Measuring spectra offers several interests. As the spectral rejection of the filters (see Methods), used to suppress the driving beams (*ω* and 2*ω*) and to isolate the studied harmonic order, is not efficient, spectra can reveal a large part of the harmonic content of the generation in two-color and single-color setups. This brings valuable information about the process of harmonic generation. Also, this information is of primary importance for the interpretation of the harmonic spatial properties since detectors are polychromatic.

**Figure 1:**
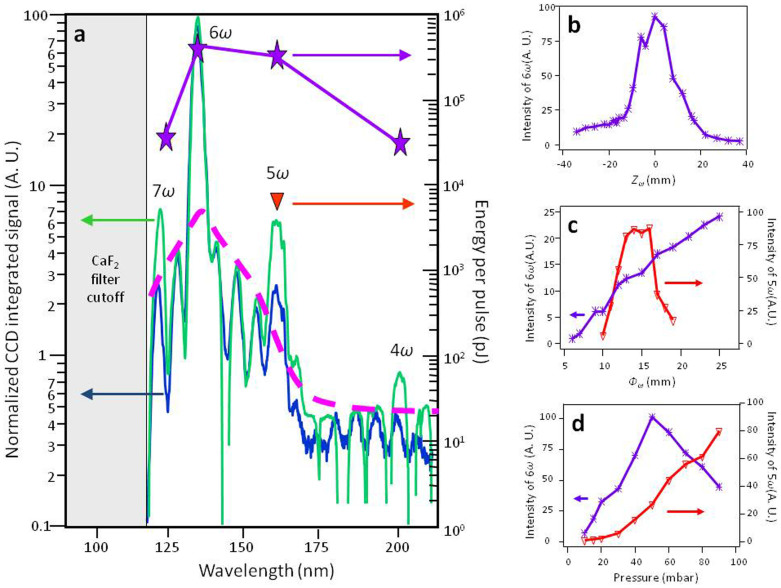
Harmonic flux. (a) Left side y-axis: normalized spectra in *ω* + 2*ω* setup with two filters (green) and three filters (blue). Due to the diffraction of the 6*ω* beam on the transmission grating grid, the effect being far to be non-negligible here for an intense low order beam, additional peaks are present in the spectra. Notably some peaks can be superimposed on existing peaks such as the ones corresponding to 5*ω* and 7*ω*. From the two spectra, a visual estimation of the maximum importance of this effect on other harmonics can be made (pink dashed line). These considerations take into account all of the diffraction peaks which do not correspond to potential harmonics. Right side y-axis: absolute energy per pulse (or per shot) in *ω* + 2*ω* (violet stars) or *ω* (red triangles) configurations. Optimization parameters: for 6*ω*, *Φ_ω_* = 25 mm, *I_ω_* = 2 × 10^14^ W.cm^−2^, *I_2ω_* = 5 × 10^13^ W.cm^−2^, *Z_ω_* = 0 and the gas pressure is 50 mbar, for 5*ω*, *Φ_ω_* = 14 mm, *I_ω_* = 1.6 × 10^14^ W.cm^−2^, *Z_ω_* = 0 and the gas pressure is 90 mbar. Evolution of the 6*ω* (violet stars) and 5*ω* (red triangles) intensities as function of (b) the longitudinal position *Z_ω_* in the gas cell of the laser focus with *Z_ω_* = 0 corresponding to the middle position and for *Φ_ω_* = 25 mm, (c) the diameter of the iris *Φ_ω_* placed in the laser beam path for *Z_ω_* = 0, and (d) the gas pressure for *Z_ω_* = 0. For 5*ω* *Φ_ω_* = 25 mm and for 6*ω* *Φ_ω_* = 14 mm.

Using two filters (see Methods), the residual laser beams are sufficiently suppressed. However, Fig. 1a shows that in two-color configuration spectra, 4*ω*, 5*ω* and 7*ω* are detected in addition to 6*ω* (green curve compared to pink fit). Deconvolving by the transmission of the filters and the theoretical transmission of the optics located in the beam path, one can access to the raw generated spectrum, which is here clearly optimized for 6*ω* (violet stars). The spectrum also shows that all even harmonics, visible in this spectral window, are generated. The presence of 4*ω* content confirms that we do observe mixing generation process spectra, 6*ω* being not the 3^rd^ harmonic of 2*ω* generated in gas.

With an additional filter, only a residual part of the 5*ω* (about 1% of 6*ω*, blue curve compared to pink fit) slightly contaminates the 6*ω* signal. In single-color generation regime, the 5*ω* signal is the only observable frequency (not shown here). Three filters (see Methods) are then employed for all the measurements in this paper. Fig. 1b–d display the evolution of 6*ω* and 5*ω* signals as function of the main identified parameters of the harmonic generation. From Fig. 1b–c, one can observe that optimal conditions of generation for 6*ω* are not attained. First, the highest available 6ω signal is obtained for a laser focusing at the center of the gas cell (*Z_ω_* = 0, Fig. 1b, violet). Second, the 6*ω* yield keeps increasing for larger iris aperture (Fig. 1c, violet), corresponding to the maximum available energy and to the smaller waist at focal spot. At contrary this is not the case for the generation of 5*ω* in single-color (Fig. 1c, red). It then clearly indicates a lack of driving laser intensities in this two-color geometry. Finally the pressure regimes for the generation are also different (Fig. 1d). As observed in ref. 20 for higher orders, the optimized pressure is at least twice smaller in *ω* + 2*ω* case.

Accounting for the efficiency of the CCD camera, the maximum available energy per pulse (or per shot) for the two main studied harmonics is estimated (see Methods) using their optimized parameters deduced from Fig. 1a and in the two filters case. In this relatively loose focusing geometry (1 m), from a 3 mJ *ω* pulse an ~0.3 µJ 6*ω* pulse can be generated, meaning an efficiency of ~10^−4^. While a similar pulse energy and efficiency can be obtained for the 5*ω* harmonic in the two-color generation geometry, they become much lower (more than 60 times) in the single-color optimized geometry (2 mJ *ω* pulse). This increase of the generation efficiency in a two-color setup confirms previous results obtained also for low order harmonics but in different conditions and especially the polarization configuration^40^. Indeed, while in ref. 40, harmonics are obtained by frequency mixing of both parallel beams, our harmonic generation configuration is quite unusual since polarizations are perpendicular to each other, a configuration for which the harmonic generation efficiency is often expected to be weak. In addition, laser properties were dissimilar, both in term of fundamental wavelength (1064 nm) and duration (35 ps)- this latter notably affecting the generation of the second harmonic by the crystal. This also verifies results obtained for much higher orders^41^,^42^ and in addition in exactly same configuration^15^.

### Spatial intensity profiles

The spatial intensity profile measurements have been performed in 0.1 s, corresponding to the average of 100 shots. They have been obtained by changing the main parameters of generation such as aperture of the iris, focusing position inside the gas cell and gas pressure. For fixed parameters, the shape of the spatial intensity profiles remains, acquisition after acquisition, almost exactly the same.

**Figure 2:**
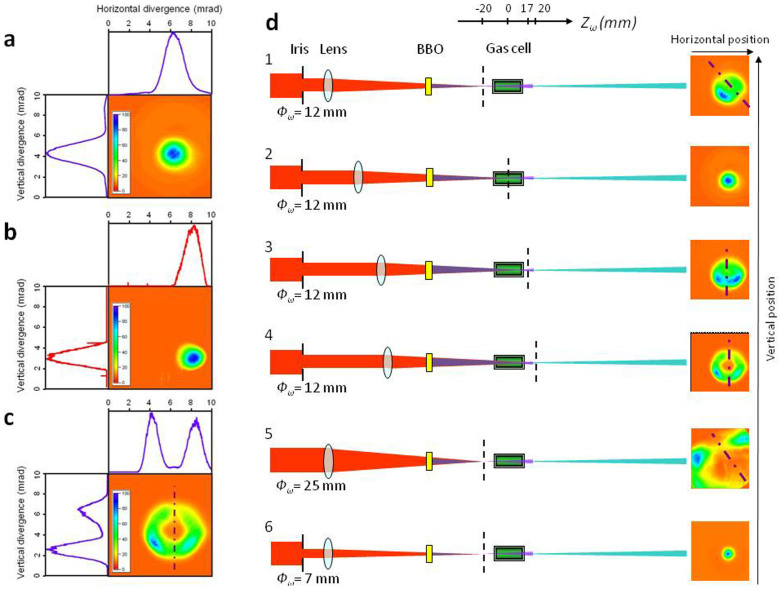
Harmonic spatial intensity profiles (false colors, normalized). (a) 6*ω* using *I_ω_* = 2 × 10^14^ W.cm^−2^, *I_2ω_* = 5 × 10^13^ W.cm^−2^, *Φ_ω_* = 25 mm and *Z_ω_* = 0 mm. (b) 5*ω* using *I_ω_* = 1.6 × 10^14^ W.cm^−2^, *Φ_ω_* = 14 mm and *Z_ω_* = 0 mm. (c) 6*ω* using *I_ω_* = 1.2 × 10^14^ W.cm^−2^, *I_2ω_* = 4 × 10^13^ W.cm^−2^, *Φ_ω_* = 12 mm and *Z_ω_* = 20 mm. (d) 6*ω* : evolutions as function of the longitudinal focusing of the *ω* beam inside the gas cell with *Φ_ω_* = 12 mm (1–4) and as function of the iris diameter placed in the *ω* beam path with *Z_ω_* = −20 mm (1, 5, 6). The black dashed line points out the longitudinal focus plan of the driving beams. The violet dashed and dotted line indicates the axis of quasi-symmetry of the non-homogeneities.

As shown in Fig. 1, the strongest 6*ω* signal is attained for the maximum laser beam diameter, focusing position inside the gas cell and a pressure of 50 mbar. In these conditions, the spatial distributions of 6*ω* (Fig. 2a) present a characteristic regular Gaussian-like shape with a divergence of about 2 mrad FWHM (Full Width at Half Maximum). The 5*ω* beam obtained in single-color (Fig. 2b) has very similar spatial characteristics. Yet, the smooth and single peaked distribution of the 6*ω* beam can vanish and turn into a non-homogenous donut shape when shifting significantly the position of laser focus from the original centered position inside the gas medium (Fig. 2c, *Z_ω_* = 20 mm, 20 mm after the center of the gas cell). In this distribution a clear quasi-symmetry of the non-homogeneities is observed, determining two lobes, and the divergence is increased at least up to 5 mrad FWHM depending on the longitudinal focusing position condition.

The evolution of this intensity profile differs if the laser is focused upstream or downstream of the gas cell center (Fig. 2d). Indeed, while for symmetric positions compared to the centered position inside the gas cell the divergence of the harmonic beam is identical and that both distributions still looks like non-homogenous donuts, these non-homogeneities are spread differently. Also, the axes of symmetry of the non-homogeneities are tilted from each other (by about 40 degrees). To better understand this evolution, the laser focus position was precisely scanned. From an extreme position to the centered position, the divergence decreases and the donut gradually collapses (keeping the same symmetry axis of non-homogeneities) into a perfect Gaussian-like distribution. The evolution of the non-homogenous donut shape has also been explored by changing the laser aperture (Fig. 2d1, 5, 6) for an inadequate focusing position (*Z_ω_* = -20 mm). From full aperture (*Φ_ω_* = 25 mm) to small aperture (*Φ_ω_* = 7 mm), the donut intensity distribution gradually evolves towards a Gaussian-like distribution (respectively Fig. 2d5 and Fig. 2d6). Finally, changing pressure of Argon inside the gas cell did not reveal any effect on the radiation profiles in any studied cases.

### Spatial coherence

**Figure 3:**
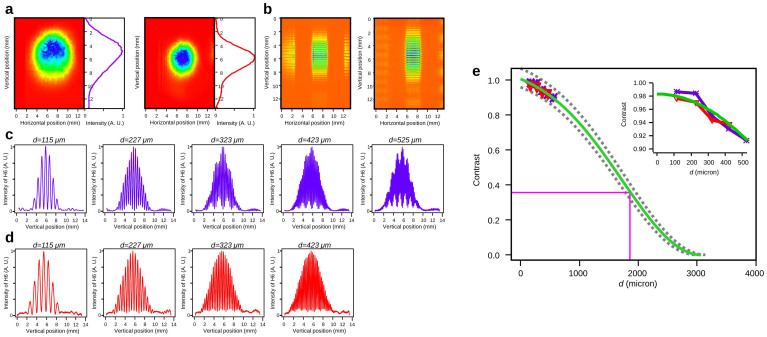
Harmonic spatial coherence measurements. (a) Spatial intensity profile of 6*ω* (normalized, violet, *Z_ω_* = 0 mm, *Ф_ω_* = 10 mm, *I_ω_* = 1 × 10^14^ W.cm^−2^ and *I_2ω_* = 1 × 10^13^ W.cm^−2^) and 5*ω* (red, *Z_ω_* = 0 mm, *Ф_ω_* = 10 mm, *I_ω_* = 1.3 × 10^14^ W.cm^−2^). (b) Interference patterns of 6*ω* (left) and 5*ω* (right). (c)–(d) Vertical cross sections for different d (distance between the slits) and respectively for 6*ω* and 5*ω*. (e) Contrast of 5*ω* (red) and 6*ω* (violet) as function of *d*, the distance between the slits. The green curve represents the best theoretical Gaussian fit of the data. The grey dotted lines correspond to the estimated realistic boundaries of this fit. The purple lines are disposed to evaluate the degree of spatial coherence. The inset is a zoom on the measurement part.

In this section, the spatial coherence of the 6*ω* beam is measured and compared to the 5*ω* one (Fig. 3 respectively with violet and red colors) using Young slit setup. For each distance (*d*-parameter) between the slits, typical interferograms (Fig. 3b), which have been obtained from far-field spatial intensity profiles (Fig. 3a), are recorded. Cross sections of these interferograms (Fig. 3c, d) are used to retrieve the spatial coherence by measuring the contrast of the interference pattern (Fig. 3e). Conditions of generation of 5*ω* and 6*ω* beams are here more or less similar; the fundamental laser beam is focused in the centre of the gas cell and is clipped to 10 mm aperture to limit the size of the harmonic beam on the slits. Indeed, due to the high divergence of the harmonic beam for low orders and due to technical limitations, notably our slits setup optimized for higher order harmonics, the size of the beam on the slit system remains much bigger than the slits separation. As a consequence, only a small part of the beam is really probed in the available range of measurements. Nevertheless, we may estimate with the five first positions (five different *d*) the trend of the contrast evolution, which is supposed to follow a Gaussian curve (Fig. 3e green curve), for a Gaussian source. The coherence length, i.e. the characteristic distance on which the electromagnetic waves of the source preserves its phase correlation^43^, corresponds to a decrease of the contrast of 1/e. In our case, the fitted curve indicates a coherence length of 1850 ± 100 µm. This precision is obtained from the boundaries of this fit, corresponding to ± 2% estimated maximum error on the determination of maxima and minima on the interference pattern. At the slit system position, the size of the beam at 1/e is estimated to be about 4500 µm for both beams, which gives a coherence percentage of the beam of 41 ± 2.5%. Compared to previous measurements^44^,^45^,^46^, this is a classical value for high harmonic generation. The contrast evolutions for 5*ω* and 6*ω* harmonics are very similar, and as beam sizes are almost identical, the coherence percentages of these two harmonics should be almost equal.

### Spatial aberrations

**Figure 4:**
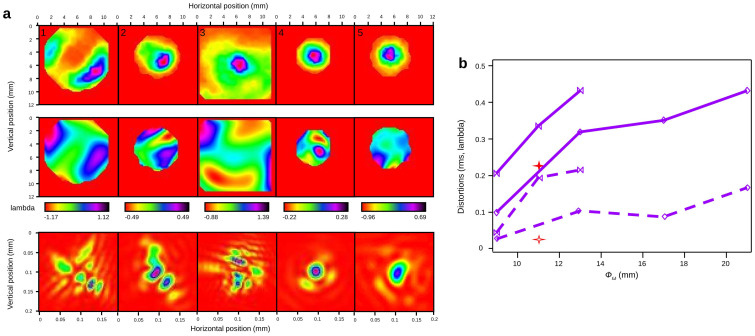
Harmonic spatial wavefront measurements. (a) Images in false color of the far-field spatial intensity distribution (normalized, up), far-field wavefront distribution (middle) and retro-propagated spatial intensity distribution at focus (down) of 6*ω* using (1) *Ф_ω_* = 13 mm and *Z_ω_* = 20 mm (2) *Ф_ω_* = 9 mm and *Z_ω_* = 20 mm (3) *Ф_ω_* = 21 mm and *Z_ω_* = 0 mm (4) *Ф_ω_* = 9 mm and *Z_ω_* = 0 mm (5) of 5*ω* using *Ф_ω_* = 11 mm and *Z_ω_* = 0 mm. (b) Wavefront aberration level for 6*ω* (violet) and 5*ω* (red) and corresponding to *Z_ω_* = 20 mm (double triangle) and *Z_ω_* = 0 mm (diamond). Plain line: total distortions (except tilt and spherical aberrations which are automatically suppressed by the software). Dashed line: only astigmatism.

Since the Hartman wavefront sensor is sensitive in a wide range of radiation wavelengths, one usually measures a composite wavefront of the polychromatic high harmonic beam^47^,^48^. Here, and to our knowledge for the first time, the real aberrations of a spectrally isolated harmonic are evaluated. Fig. 4a displays for different configurations of laser apertures and focusing positions inside the gas cell, the measured spatial intensity distributions of the harmonic in the far-field (up), the corresponded wavefront pattern (middle) and the calculated spatial distributions at the focus inside the gas cell (down). This calculation is obtained by retro-propagation using a software developed by Imagine Optic^49^ which takes into account information about intensity and phase delivered by the wavefront measurement. The rms (root min square) distortion level is displayed in Fig. 4a with a wavelength unity scale (not nm) for representing the spatial variations inside the beam and in Fig. 4b with averaged values of the spatially integrated beam.

When the fundamental beam is focused 20 mm after the gas cell center (Fig. 4a1, 2), the 6*ω* spatial intensity profile exhibits a donut shape with two main lobes, as seen previously. The corresponding wavefront pattern also shows this structure, with highest aberrations actually located in these lobes. Distortions of the wavefront reach values, from *λ*/2.5 rms to *λ*/5 rms (*λ = *135 nm) for apertures respectively of 13 mm (Fig. 4a1) to 9 mm (Fig. 4a2). In the first case, with this relatively high distortion the calculation of the spatial distributions at focus is complex and it shows complicated structures (Fig. 4a1-down). In the second case with smaller distortions, the two-lobe shape of 6*ω* at focus can be evidenced (Fig. 4a2-down). For optimal focusing inside the gas cell (Fig. 4a3) and with 21 mm iris aperture the level of distortions reaches *λ*/2 rms (the distortions being mainly located in the outer part of the beam). It almost decreases to *λ*/10 rms with 9 mm aperture (Fig. 4a4) and the corresponding waist at focus exhibits an almost perfect Gaussian-like shape. In all the studied cases, the major part of the distortions (about 50%) measured on the 6*ω* beam originates from astigmatism. In addition, the distortions of the 5*ω* have also been evaluated to be about *λ*/5 rms (*λ = *160 nm, Fig. 4a5, and Fig. 4b red cross for aperture of 11 mm) for its optimal generation conditions. For comparable conditions, the distortions of 6*ω* seem to reach the same value or even lower as seen in ref. 47, but the statistic of this measurement is here restricted to clearly establish it.

In conclusion, even by focusing at the perfect position inside the gas cell, for a full laser aperture, aberrations, yet minimized, are still visible in the wavefront pattern. To get a quasi diffraction-limited harmonic beam, the laser beam has to be strongly clipped, reducing the energy per pulse by a factor of 5 (Fig. 1c). Of course, aberrations from the fundamental laser should be reduced down to the minimum upstream to improve the spatial intensity distribution of the harmonic, to decrease its distortions and to increase the generation efficiency.

## Discussion

Donut-like shapes, such the one observed in our case for the spatial intensity profile of the 6*ω* beam, have already been reported in literature. Yet, their origins are deeply different. First, Salieres et al.^50^ showed theoretically and experimentally spatial distributions of harmonics at high orders that revealed to be in some cases annular. Changes of the shape and important increase of the divergence of the harmonic occurred with the position of the laser focus compared to the medium (in their case a gas jet and not a gas cell). Their results have been obtained at much higher orders and for different laser conditions since the intensity of the driving beam was above the ionization potential of the atoms. In this case, the interplay between short and long electron trajectories plays a major role. Phase matching variations -either on-axis or off-axis- in the nonlinear medium depending on the dipole phase gradient direction then favor one of the distribution. A second observation allows in principle to exclude this phase-matching effect, or at least to show that it is not dominating. Indeed, the longitudinal range over which the shape turned into annular was quite different. In spite of the fact that they used a lens with a slightly shorter focal length of 0.75 m (compared to 1 m), the variations of the shape were observed within a very short distance of couple of millimeters (compared to 20 mm). More recently, a theoretical article showed annular distribution of harmonics in the supercontinuum generated also in two-color configuration^51^. As for ref. 50, the observed harmonics are generated differently and especially at much higher orders. The annular behavior was explained to be caused by the shape at the focus inside the medium of the 2*ω* component. The spatial distribution of the field either favors on-axis or off-axis emission depending on the position of the focus and of the waist size, privileging different phase matchings. In addition, a large number of harmonic orders (from 40^th^ to 60^th^) were integrated and as a consequence effects of both odd and even order were mixed together.

The non-homogeneous donut shape turns into a Gaussian one when either focusing the fundamental laser beam at the center of the gas cell or when clipping this beam. This might indicate that the complex shape is related to the aberrations of the fundamental laser. The effect of these aberrations would not be easily visible when observing profiles obtained with accurate focus at the center of the gas cell. Also, decreasing the beam aperture would allow to reduce the aberration level that is known to strongly increase on the beam edges. The origin of the non-homogeneous donut shape would then come from the presence of the BBO crystal as this phenomenon is not observed in the single-color setup. In ref. 52, it is reported that aberrations from the fundamental laser beam, i.e. variations of the spatial phase in the transverse plane of a doubling crystal, can lead to altered phase-matching conditions. They give rise to a spatially non-homogenous generation of the second harmonic. Depending on the type, the strength and the distribution of these aberrations a complex final shape can emerge from that. In the two-color setup, harmonics are generated where both beam intensities are high enough. In our geometrical configuration, both beams are focused at the same position but the second harmonic is focused in a smaller waist (due to the shorter wavelength). Consequently, the 2*ω* shape at the focal position inside the gas cell should principally contribute to the 6*ω* profile in the far-field as said by ref. 51.

**Figure 5:**
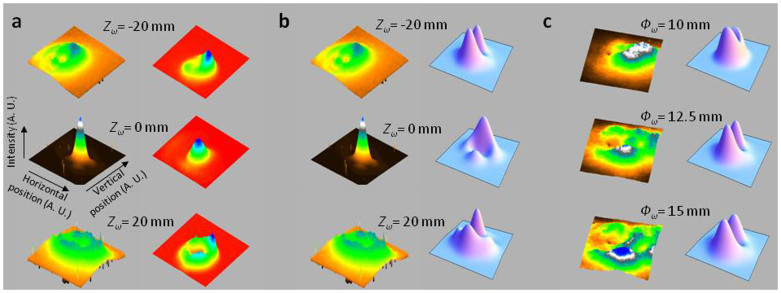
Comparisons of experimental and simulated spatial intensity profiles of 2*ω* and 6*ω*. (a) Measured spatial intensity distributions (normalized) of 2*ω* (right, near-field) and 6*ω* (left, far-field) as function of the position of the longitudinal focusing of the laser inside the gas cell. *Φ_ω_* = 12 mm, *I_ω_* = 1.4 × 10^14^ W.cm^−2^ and *I_2ω_* = 1.2 × 10^13^ W.cm^−2^. (b) Near-field spatial intensity distributions (false color, normalized). Theoretical 2ω (right) and experimental 2*ω* (left) as function of the position of the longitudinal focusing of the laser inside the gas cell. *Φ_ω_* = 12 mm. (c) Near-field spatial intensity distributions (false color, normalized). Theoretical 2*ω* (right) and experimental 2*ω* (left) as function of the laser aperture. *Z_ω_* = −20 mm.

To support this idea, we have measured the spatial intensity profile of the second harmonic in a plane located at the central position of the gas cell and for different positions of the laser focus compared to this position. These second harmonic profiles are then compared to 6*ω* profiles obtained in the same conditions but in the far-field (about one meter away from the gas cell center) in Fig. 5a. Both harmonic profiles look qualitatively similar. Indeed, for same laser focusing positions, donut shapes with same non-homogenous distributions and same axis orientations of the non-homogeneities are observed. When focusing at the center of the gas cell, purely Gaussian distributions can also be seen for both beams. Propagation effects from near-field to far-field on the 6*ω* beam seem therefore to weakly modify the intensity profiles. A proof of that is given by Fig. 4a2, showing the retro-propagated 6*ω* spatial intensity profile in the near-field (down) with quite similar two-lobes distribution and same orientation of the symmetry axis of this distribution, as the corresponded far-field ones (up). In conclusion, primary evidences that the non-homogeneous donut shape is originating from laser aberrations have been brought, indicating in addition that the second harmonic profile imprints significantly the shape of the 6*ω* profile.

Knowing that the main part of the distortions of the 6*ω* beam is astigmatism, we may reasonably expect that the aberrations come directly from the fundamental laser itself. It would cause the altered phase-matching conditions inside the BBO crystal leading to the generation of spatially non-homogeneous second harmonic intensity profiles and then to the observed complex shapes of the 6*ω* spatial intensity profiles. To definitively validate our assumption, we have simulated (see Methods) the spatial intensity distributions of the 2*ω* beam and compared them to the measured ones when changing the fundamental laser aperture and laser focusing position inside the gas cell (Fig. 5b, c). They also show complicated structures, which are very similar to the non-homogeneous donut. Also, when increasing the distance to the focus (b) and the aperture (c), the complicated structures get more prominent. Additional simulations (not shown here) pointed out that complex distributions can also be obtained through coma type aberrations from the fundamental laser. The shape, that we have observed experimentally (Fig. 2c), should then originate from a combination of effects of different aberrations, the one of astigmatism being here dominant. In the future, we plan to implement a more elegant technical solution, allowing to directly measure the wavefront of the fundamental beam, for retrieving properly the different types of aberrations and simulating more accurately the second harmonic profile.

The longitudinal range, for which the harmonic spatial intensity profile is regular and quasi-Gaussian, is about 20 mm: 10 mm upstream and 10 mm downstream the gas cell center. This range is experimentally very easy to be targeted. Compared to our 1 m focal length this corresponds to ±1% error in the laser focusing position. Even for very short focal lengths one can avoid this undesirable spatial effect, without any difficulty, by focusing properly the laser with mm precision. Independently of that, aberrations of the fundamental laser can be decreased significantly by different ways in order to observe harmonic beams with Gaussian spatial distribution. The first technique, shown in this paper, consists in clipping the beam. Yet, as seen in previous references and in this paper, even if it is technically easy to be performed, it brings non trivial results in term of harmonic efficiency. Indeed, modifications of the beam spatial structure engender important changes of fundamental parameters (mainly Gouy phase, intrinsic phase, and density of the free-carriers) of the harmonic generation process. While in some cases, the clipping can allow to improve the efficiency^53^,^54^, in our case, as said previously, it causes an important drop of this latter due to a lack of driving laser intensities in our two-color geometry. Upstream of the laser chain several other modifications can be made. With typical solid-state lasers such as our Ti: Sa, one can improve the spatial qualities of the pump beams, which are focused on the crystal. Generally, using optical elements with the best quality and/or bigger size, is also requested. In the last resort, adaptive optics^55^ can be implemented in the laser line together with Infra-Red wavefront sensor to compensate efficiently the aberrations and get purely Gaussian spatial 6*ω* harmonic beam shape. Changes of the types of aberrations of the fundamental laser and strengths of them can be performed accurately allowing, when measuring in parallel the wavefront profile of the harmonics and of the fundamental laser, to really evaluate the “transmission” of the aberrations. One can envisage also to appreciate in several aberration cases the variations of the second or 6*ω* harmonic profile. Yet, these modifications of the aberrations can in reverse modify the efficiency of the harmonic generation process. Indeed, non-aberrant laser beams in general allow to generate harmonic beams with an aberration level at minimum and a perfectly Gaussian spatial distribution but with most of the time a lower energy per pulse^56^.

In summary, we have shown for the first time a detailed study of spectrally isolated low order harmonics. This spectral region is of high interests for many applications, requiring in any case both high intensity and high spatial quality. We have analyzed and distinguished the behaviors of the generation of an even order harmonic 6*ω* (obtained in a two-color setup) and of an odd order harmonic 5*ω* (single-color setup). The efficiency of the generation of the 6*ω* harmonic at 135 nm was estimated to be close to 10^−4^, about 60 times larger than the one of the 5*ω* harmonic at 160 nm. This was obtained by focusing, at the center of a gas cell filled with Argon at 50 mbar pressure, using almost full laser aperture (about 3 mJ energy per pulse) to extract as much as possible energy. The spatial quality of the even harmonic was proved to be at least as good as for conventional harmonics, through the measurements of the spatial intensity profiles, the spatial coherences and the spatial aberration profiles. We have also clearly established some effects of the fundamental laser aberrations on the selected even order harmonic. Distortions from the fundamental laser, here strong astigmatism, can induce a non-homogenous donut shape on the 2*ω* spatial intensity profile at focus inside the gas cell, this shape being then imprinted on the 6*ω* spatial intensity profile. This non-homogenous spatial effect can be easily suppressed by either focusing correctly the fundamental laser beam inside the gas cell, or clipping significantly the laser beam. Requirements are even stronger for cancelling the main part of the harmonic distortions since both perfect focusing (center of the gas cell) has to be combined with large clipping of the laser beam (9 mm laser aperture is necessary for 25 mm full laser aperture). Implementation of adaptive optics should be then envisaged. In conclusion, this study showed the primary role of the 2*ω* beam in the generation of even order harmonics and the importance of controlling accurately both *ω* and 2*ω* spatial properties in view of applications.

## Methods

### Experimental parameters

**Figure 6:**
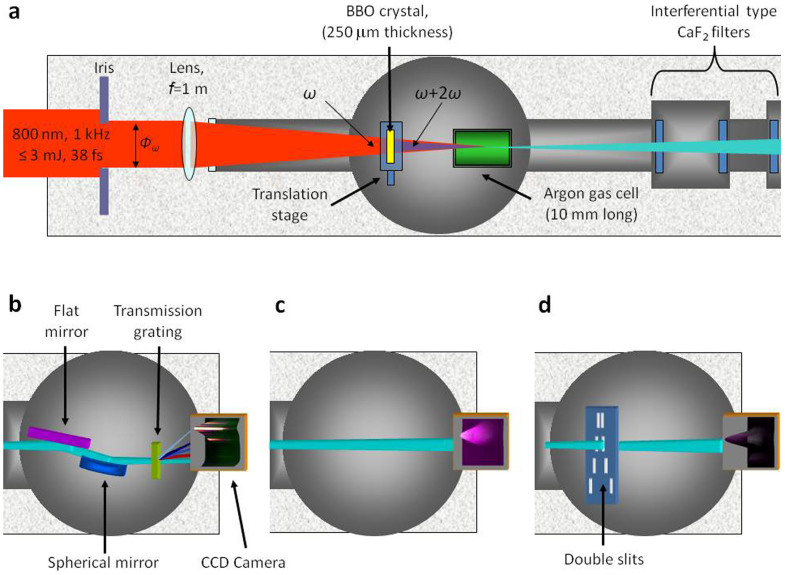
Top views of the experimental setup. (a) First part: laser shaping and harmonic generation in *ω* + 2*ω* case. *f* is the focal length of the lens and *Φ_ω_* is the aperture of the iris disposed on the fundamental laser path. (b)–(d) Second part: measurement systems of respectively spectrum, spatial intensity and/or wavefront profiles and spatial coherence.

The experiment has been performed at the Laboratoire d'Optique Appliquée (France) by means of a kHz Ti:Sa laser system at 800 nm (*ω*) delivering maximum of 3 mJ energy in 38 fs FWHM pulses. Fig. 6a presents a schematic description of the harmonic generation experimental setup.

In order to generate the second harmonic (2ω), a 250 µm thickness BBO (Beta Barium Borate, type 1) doubling crystal, is directly inserted in the ω beam path between a 1 m focusing lens and a gas cell (10 mm long) filled with Argon (at relatively high pressure: > 50 mbar). In this geometry, the 2ω beam propagates along the same axis as the ω beam. Consequently, both beams are focused at the same longitudinal position in the active medium and are automatically spatially overlapped. Limiting the crystal thickness to less than 300 µm, the temporal delay between the two pulses, induced by the dispersion of the crystal, is small and the temporal overlap is ensured [This result has been obtained by calculations using the SNLO (Select NonLinear Optics) software]. By removing the BBO crystal (while under vacuum), single-color harmonic generation can be obtained with minimal intervention. Yet, in these two configurations, intensities interacting into the gas medium can be significantly different. Indeed, in the two-color case due to losses in the second harmonic generation process (reflection, absorption from the BBO crystal and conversion to 2ω) the maximum available fundamental laser intensity is lower than in single-color case. In addition, as short BBO crystal thickness and relatively low fundamental energy per pulse are employed here, the intensity of the second harmonic is restrained owing to a moderate efficiency conversion (5 to 10%).

Various parameters can be changed when optimizing the harmonics, like gas pressure, position of the laser focal spot referred to the gas cell (*Z_ω_*), aperture of the iris disposed on the *ω* beam path (*Φ_ω_*) and intensity of the *ω* beam (*I_ω_*). In our setup aperture and intensity of the fundamental beam are linked to each other since clipping the beam reduces the intensity. The aperture also affects the intensity of the second harmonic (*I_2ω_*). The argon gas cell interacts with the laser at intensities of about 2 × 10^14^ W/cm^2^ for the fundamental laser and 5 × 10^13^ W/cm^2^ for the second harmonic.

### Spectral filtering

Getting 2D spatial information of spectrally isolated harmonics is not a trivial measurement, since technically the possibilities to select harmonics are not numerous. For high order harmonics, filters are not selective enough and therefore multilayer mirrors with narrow band but low reflectivity have to be added, allowing in best cases to observe only a few harmonics mixed together. This becomes even worst in a two-color setup as even harmonics are also present. Associating focusing optics and gratings allows to separate correctly the different orders, but this usually introduce a lot of aberration and generally one can only get information in the vertical dimension of the beam^57^. One quite elaborated solution could be that of selecting the desired harmonic by a second mechanism such as amplification in plasma based soft X-ray laser^58^ or FEL^59^, but in these cases the shape of the amplified harmonics would be largely dependent of the amplification medium. In summary, for high orders it is almost impossible to clearly distinguish behaviors of odd and even harmonics generation. At low orders, simple interferential filters enable the measurements of each contribution separately. Several interferential calcium-fluoride filters, with a band pass centered at 135 nm, have then be inserted in the optical path in order to block the driving beams (*ω* and 2*ω*) before the diagnostic vacuum chamber. These filters can be narrow band type (+/− 2 nm of bandwidth at FWHM); the rejection per filter of the surrounding harmonics and driving beams is yet partial and consequently three of them are needed to efficiently isolate either 6*ω* in the two-color case or 5*ω* in the single-color case. Broadband type filters (+/− 10 nm of bandwidth at FWHM) can also be used; they allow residual transmission at other low-order harmonic radiations. To acquire spectra, a combination of one narrow band filter and one broadband filter has been chosen. All filters have been calibrated in-situ. Basically, the transmission per narrow band filter is about 4% at 160 nm and 10% at 135 nm. For broadband type, the transmission per filter is about 23% at 160 nm and 37% at 135 nm.

### Measurements diagnostics

In the diagnostic chamber, spectrum (1), spatial intensity (2) and/or wavefront (3) profiles, and spatial coherence (4) can be measured alternatively.

1. The spectrometer (Fig. 6b) is composed by a spherical mirror placed at 15° grazing incidence (50 cm focal length) with 800 nm anti-reflection coating (ZrO_2_/Si multilayer), a transmission grating (1000 lines/mm) and a back-illuminated CCD Camera (Princeton, 1024 × 1024 pixels, 13 × 13 μm^2^). Acquired pictures represent in vertical direction the vertical distribution of the beam and in horizontal the wavelength of emission. Spectra are then obtained by the integration over the vertical dimension. To determine the absolute energy per pulse of harmonic photons in the different configurations of generation and for the different harmonic orders, each optical element has been experimentally characterized in the spectral range of the experiment. Indeed, the reflectivity of the spherical grating and the efficiency of the grating has been evaluated independently, using various filter types and filters optimized at different wavelengths. These values have been correlated to tabulated data, accounting for material composition and angles of incidence. As said previously, each filter transmission has been calibrated in situ. Concerning the CCD camera, the efficiency has been determined from technical data provided by the company selling the camera itself.
2. The spatial intensity distributions are obtained by simply letting the beam diverge up to the CCD camera (Fig. 6c).
3. The wavefront distortions are measured along the axis of the harmonic emission with a soft X-ray Hartmann type sensor (Fig. 6c), developed in collaboration with Imagine Optic firm^49^. The beam passes through an array of equidistant small apertures, the diffracted beamlets being projected onto the CCD camera. The position of each individual spot centroid is measured and compared with a reference position corresponding to a beam not affected by aberrations. This enables the wavefront local slope to be evaluated for a large number of points all over the beam aperture, allowing the wavefront to be precisely reconstructed. Then both spatial intensity and wavefront profiles can be mapped in a single measurement. This diagnostic allows the reconstruction of the wavefront with a resolution of *λ*/20 rms at 32 nm.
4. The spatial coherence is evaluated by introducing in the beam pass a double-slits system with variable distance between each other (Young's slit technique), the induced interference patterns being then acquired on the CCD camera (Fig. 6d).

### Simulation details

Using an analytical code in accordance to ref. 52, spatial distributions of the second harmonic have been here simulated for this main aberration type -astigmatism- neglecting all the other aberration types. The field of the fundamental laser is modeled by the following way in the cylindrical coordinates (*r,θ,z*): 
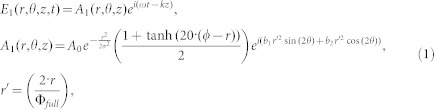
Where *A*_0_, *ω* and *k* are respectively the amplitude, frequency and wave-vector of the fundamental laser. *σ* is the rms spatial parameter of the fundamental laser beam assumed to have Gaussian distribution and with value is determined from the diameter of the laser beam before being clipped by the iris (Ф*_full_*~25 mm). The functions 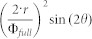 and 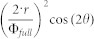 correspond respectively to the Zernike polynomial terms defined in the full aperture of the laser beam describing third-order astigmatism at 45° and astigmatism at 0° degree. The coefficients *b_1_* and *b_2_* represent the strength of these components. The function 0.5(1 + tanh(20·(*ϕ* − *r*))) models the modification of the beam structure due to the presence of the iris with *ϕ* being its radius.

Using the equations (1) the profile of the incident fundamental laser field can be easily calculated at the entrance plane of the BBO crystal (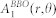) accounting for the propagation of the laser field inside and after the lens. Then, the process of second harmonic generation can be described with the help of coupled equations for the slowly varying amplitudes: 
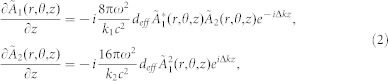
Where 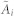 and *k_i_* (*i* = 1,2) are respectively the complex amplitudes and wave-vectors of the fundamental and the second harmonic inside the BBO, Δ*k* = 2*k*_1_ −*k*_2_ and *d_eff_* = 4.3·10^−9^ for *oo* → *e* type 1 phase matching in BBO crystal (in CGS units). c is the velocity of the light. The stars “*” denote the complex conjugate.

By using the initial conditions 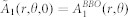, *Ã*_2_(*r*,*θ*,0) = 0 and assuming Δ*k* = 0 the set of differential equations (2) can be solved and the spatial profile *Ã*_2_(*r*,*θ*,250 *μ*m) of the second harmonic at the exit plane of the 250 µm thickness BBO crystal can be obtained.

Finally, the second harmonic field is propagated to the center of the gas cell, corresponding to the position where the experimental data have been acquired. Varying the distance between the BBO crystal and the lens as well as the iris aperture, the spatial profile of the second harmonic intensity is calculated using *b_1_* = −0.8, *b_2_* = 0.8. *b_1_* and *b_2_* have been evaluated with the wavefront sensor. These theoretical spatial intensity profiles are then compared to the experimental profiles in Fig. 5b, c.

As just demonstrated the developed model fits quite well the experimental data, even if effects occurring in thick nonlinear crystals, for which the wave equations should include the second spatial derivatives^52^, have been ignored. The model has been also tested by comparing calculated and experimentally-measured spatial intensity profiles of the fundamental laser as they should be in the single-color configuration. For various conditions of laser aperture and focusing position, good agreements have been found. Especially in any case a change of these parameters could not bring a non-homogeneous donut shape. In addition, we have calculated the spatial intensity profile of the second harmonic without accounting of any fundamental laser aberration; a Gaussian distribution has been obtained as expected.

## Author Contributions

G.L., A.P. and L.G. conducted the experiment and analyzed data. G.L. and L.G. proposed the concept of the measurements. B.V., F.T. and J.G. helped in the experimental design. G.L., L.G., P.Z., S.Se. and V.M. supervised the project. S.St. and A.A. conducted theoretical investigations leading to presented simulations. All authors discussed and contributed to the manuscript.
